# Machine learning algorithms to predict epidural-related maternal fever: a retrospective study

**DOI:** 10.3389/fphar.2025.1614770

**Published:** 2025-06-11

**Authors:** Xiaohui Guo, Haixia Zhang, Hongliang Mei

**Affiliations:** ^1^ Department of Pharmacy, Nanjing Drum Tower Hospital, Affiliated Hospital of Medical School, Nanjing University, Nanjing, Jiangsu, China; ^2^ Department of Pharmacy, Hainan Women and Children’s Medical Center, Haikou, Hainan, China; ^3^ China Hospital Reform and Development Research Institute of Nanjing University, Nanjing Drum Tower Hospital, Nanjing, China; ^4^ Nanjing Medical Center for Clinical Pharmacy, Nanjing, Jiangsu, China

**Keywords:** epidural-related maternal fever, machine learning, predictive model, Nomograms, risk assessment

## Abstract

**Introduction:**

The epidural-related maternal fever (ERMF) induced by patient-controlled epidural analgesia (PCEA) remains unpredictable. Our objective is to develop ERMF prediction models using real-world data, aiming to identify pertinent contributing factors and support obstetricians in making personalized clinical decisions.

**Methods:**

Women who used patient-controlled epidural analgesia between October 2021 and March 2023 at a tertiary hospital in Jiangsu Province were retrospectively documented. The primary outcome was the occurrence of maternal fever associated with epidural use. We developed six machine learning (ML) models and assessed the area under curve (AUC) for characteristics of subjects' performance, calibration curves, and decision curve analyses.

**Results:**

A total of 1,492 women were enrolled, with 24.3% experiencing ERMF (362 cases). The AUC ratios between the logistic regression (LR) model and the stochastic gradient descent (SGD) models showed statistical significance (p < 0.05), while the differences between the other models were not statistically significant. In comparison to the SVM model, the LR model exhibited better calibration (Brier score: 0.193; calibration slope: 0.715; calibration intercept: 0.062). Consequently, the LR model was selected as the prediction model. Furthermore, the LR-based nomogram identified eight significant predictors of ERMF, including neutrophil percentage, first stage of labor, amniotic fluid contamination during membrane rupture, artificial rupture of membranes, chorioamnionitis, post-analgesic antimicrobials, pre-analgesic oxytocin, post-analgesic oxytocin, and dinoprostone suppositories.

**Conclusion:**

Optimally applying logistic regression models can enable rapid and straightforward identification of ERMF risk and the implementation of rational therapeutic measures, in contrast to machine learning models.

## 1 Introduction

Epidural analgesia (EA) has gained widespread acceptance, chosen by a significant proportion of women in labor, ranging from 10% to 83% over the past 2 decades (Seijmonsbergen-Schermers et al., 2020). Patient-controlled epidural analgesia (PCEA) is commonly used for managing labor pain. However, recent years have raised increasing concerns about the safety of PCEA, with particular attention to epidural-related maternal fever (ERMF) (Sultan and Segal, 2020). ERMF, marked by a temperature of ≥38°C, occurs in approximately 11%–33% of women who use PCEA (Sultan et al., 2016; Del Arroyo et al., 2019). This condition can disrupt obstetric management during labor, potentially necessitating additional antibiotics or a cesarean section. Moreover, it may lead to adverse neonatal outcomes, including neonatal infections, brain damage, and long-term learning disabilities (Impey et al., 2008; Ren et al., 2021). Therefore, early prediction of ERMF is of utmost importance.

Nevertheless, the etiology of ERMF remains elusive, and previous studies have identified predictors of ERMF in patients receiving PCEA. women who developed intrapartum fever had a significantly longer first stage of labor and a higher incidence of cesarean section, assisted vaginal delivery, intra-partum hemorrhage, and amniotic fluid clouding (Impey et al., 2008). Risk factors for epidural fever include prolonged labor, a low parity, ≥7 cervical examinations, higher EA dosages, extended EA duration, and total labor duration (Wang et al., 2023). Women with placental neutrophilic inflammation displayed significantly higher fever rates (Sharma et al., 2014). However, all predictors in these studies depended on traditional statistical methods, overlooking possible nonlinear relationships or multicollinearity among variables. Hence, it is vital to underscore that, even when accounting for all predictors, the method lacks a firmly established accuracy in perfectly predicting the onset of ERMF.

In the era of continually accumulating patient clinical data, contemporary healthcare decision-making demands precise, innovative, and prediction-driven decision support. Machine learning (ML) algorithms, as a statistical methodology, offer advantages over traditional statistical modeling methods due to their capacity for generalization and differentiation. Machine learning models excel at extracting implicit information from extensive datasets, potentially addressing challenges associated with using traditional logistic regression models to resolve controversies related to influential factors (Goecks et al., 2020).

Artificial intelligence (AI) is widely employed in healthcare research, and real-world studies involving machine learning algorithms have shown promising performance. AI has found application in areas like prognostic assessment for disease treatment and histologic imaging for disease diagnosis (Roozbeh et al., 2025; Banaei et al., 2025; Darsareh et al., 2023). However, models using ML algorithms to predict the influencing factors of ERMFs have not been developed yet. Consequently, this study aims to construct a predictive model for ERMF based on real-world data with the goal of identifying relevant influencing factors and assisting obstetricians in making personalized clinical decisions.

## 2 Methods

Study Design: The study was approved by the Medical Ethics Committee of the Affiliated Drum Tower Hospital of Nanjing University Medical School (approval number 2022–106-04). Written informed consent was obtained from patients or their representatives for participation in this study.

Patients: We conducted a retrospective analysis of 1,492 women who received PCEA between October 2021 and March 2023 at a tertiary care hospital in Jiangsu Province. The inclusion criteria were as follows (Seijmonsbergen-Schermers et al., 2020): Women aged over 18 and up to 50 years (Sultan and Segal, 2020); Singleton pregnancies with vaginal delivery; and (Sultan et al., 2016) Meeting the clinical criteria for administering analgesia compatible with PCEA (Laboring women requesting analgesia without the following exclusion criteria may receive PCEA: Patient refusal, Mental illness, Severe psychological distress, Spinal trauma or deformity, Puncture site infection or sepsis, Hemodynamic instability, Coagulation disorders.). The exclusion criteria were as follows (Seijmonsbergen-Schermers et al., 2020): Body temperature ≥37.5°C before PCEA (Sultan and Segal, 2020); PECA after the cervix has opened to 10 cm (Sultan et al., 2016); More than two features were missing in a single sample (Del Arroyo et al., 2019); More than 20% missing values for a Individual features.

Data collection and definitions: The primary outcome of this study was defined as the occurrence of fever (≥38 °C) in perinatal women following PCEA (3). Fever data were retrieved from nursing records, considering the highest temperature reading in cases of multiple recordings. Patient demographic information, clinical data, and laboratory test results were obtained from the electronic healthcare information system. All data underwent manual processing and verification, with missing values imputed using the K-nearest neighbor imputation (KNNI) method, which calculates the average of the K nearest eigenvalues to fill in missing data points based on eigenvalues in proximity.

Before applying machine learning algorithms, features were normalized through mean-centering and scaling to unit variance. Variable selection followed a rigorous methodology, involving a systematic review of relevant studies and expert consultations to ensure comprehensive coverage. The study involved 45 variables, categorized as five clinical variables (age, BMI, parturition, gestational week, and cervical dilatation at the onset of analgesia), 11 disease variables, 10 surgical variables, two biochemical criteria, and 27 medication-related variables. Relevant demographic information encompassed age, height, weight, BMI, gestational week, primiparity, transplacental delivery, fetal weight, and comorbid conditions such as hypertension, diabetes mellitus, Hypothyroidism, chorioamnionitis, Group B *Streptococcus* colonization, acute chorioamnionitis, and hepatitis B in pregnancy. Records of medication administered both before and after PCEA initiation included oxytocin, antibiotics (cephalosporin antibiotics), pethidine, diazepam, nifedipine, dinoprostone suppositories, misoprostol tablets, magnesium sulfate, and others. Laboratory findings obtained immediately before PCEA initiation included white blood cell count and neutrophil percentage. Additional clinical data encompassed factors such as cervical dilatation, number of vaginal examinations, artificial rupture of membranes, balloons for uterine cervical ripening, first stage of labor, second stage of labor, third stage of labor, total duration of labor, amniotic fluid contamination, Time between rupture of fetal membranes and labor onset, Intrapartum hemorrhage, etc.

Model development: The dataset was divided into training and test sets using an 8:2 ratio for model development. We applied the least absolute shrinkage and selection operator (LASSO) algorithm to reduce data dimensionality. Furthermore, all features selected by LASSO were incorporated into six models to evaluate ERMF. Model construction for machine learning algorithms was carried out using Python’s scikit-learn library, an open-source Python package specifically designed for predictive model building in machine learning. The scikit-learn package facilitates the construction of supervised machine learning models, encompassing techniques such as logistic regression (LR), support vector machines (SVM), stochastic gradient descent (SGD) (Nguyen et al., 2020), random forest classifiers (RFC) (Lai et al., 2023), random forest (RF) (Rigatti, 2017), extreme gradient boosting (XGboost) (Nwanosike et al., 2022) and multi-layer perceptron (MLP). The data from the split training set was utilized to build the machine learning algorithm model with the application of 5-fold cross-validation. Hyperparameters employed in model construction were established through grid search with 5-fold cross-validation. The algorithms were implemented in Python version 3.7, making use of the scikit-learn library version 0.24.1 and the XGBoost library version 1.2.1.

Model Evaluation and Feature Importance: Following model acquisition, we assessed predictive performance using the area under curve (AUC) score derived from the receiver operating characteristic curve (ROC) (Mandrekar, 2010). This score depends on sensitivity and specificity measurements at different thresholds, typically ranging from 0.5 to 1. A value approaching one indicates superior model performance. The AUCs and their corresponding 95% confidence intervals (CIs) were computed for each of the six models. The ROC-AUCs were statistically compared for significance using the DeLong test in R software, with a p-value below 0.05 indicating a significant difference in model performance. Additional evaluation metrics for model performance included accuracy, precision, and recall.

The calibration of the ML model on the test set was assessed by calculating the Brier score, calibration slope, and calibration intercept. The Brier score quantified the difference between the estimated and observed risk of ERMF, and models with a calibration slope of one and calibration intercept of 0 indicated perfect calibration (Oosterhoff et al., 2022). Decision curve analysis was employed to evaluate clinical utility, which considers a weighted average of true positives and false positives, by calculating net gains across a range of threshold probabilities.

Statistical analyses: The nomogram was constructed using a fitted LR model, employing the rms package. All analyses were performed using SPSS version 26.0. Descriptive statistics were applied to all variables. Subsequently, univariate analyses were carried out using the t-test for continuous variables with a normal distribution and Mann-Whitney U-tests for those with a non-normal distribution. Categorical variables were evaluated using either Fisher’s exact test or the χ2 test. All tests were two-sided, and p-values below 0.05 were deemed statistically significant.

## 3 Results

### 3.1 Study population

**FIGURE 1:**
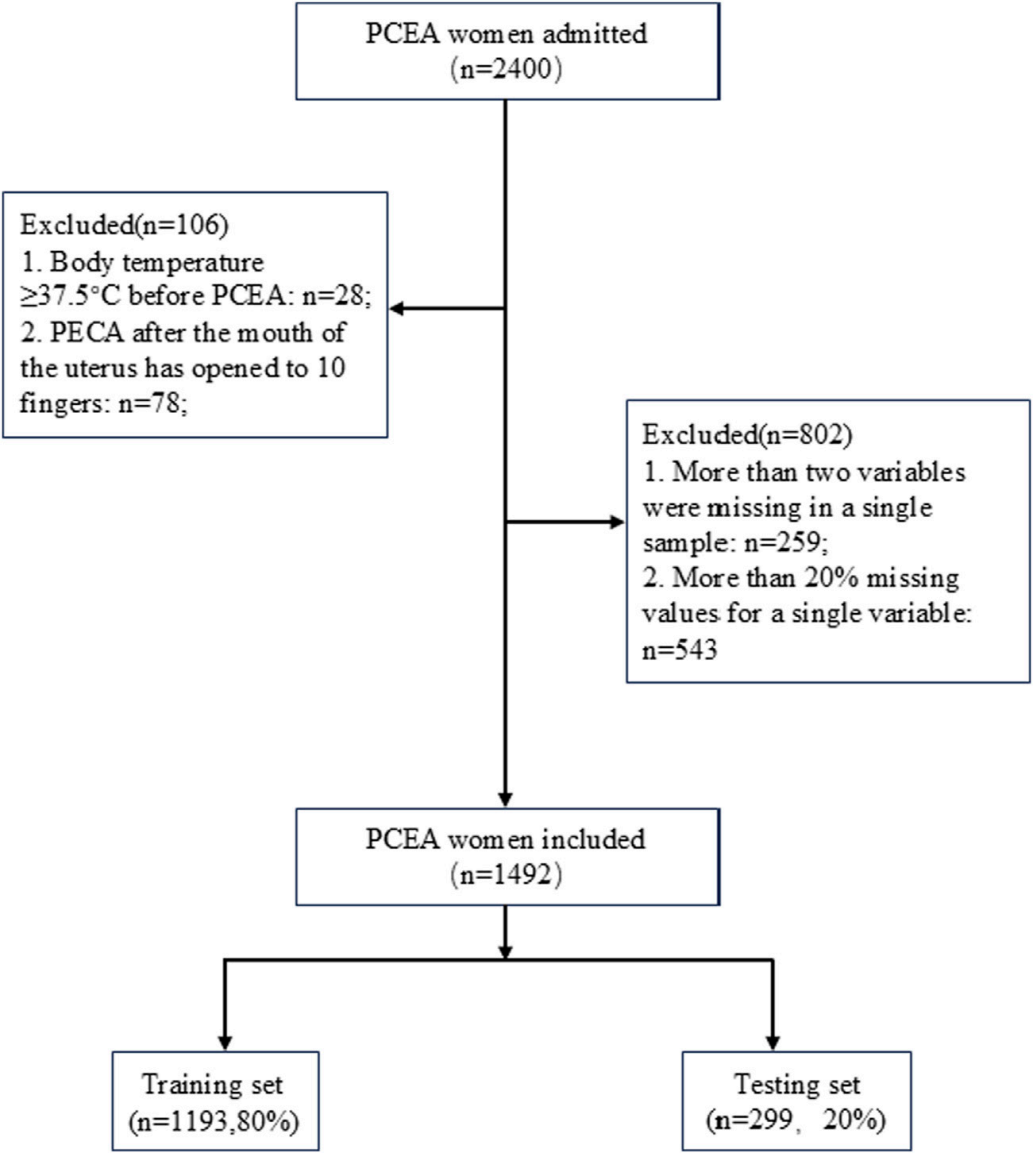
Flow chart illustrating patient selection.

Figure 1 illustrates that 1,492 patients met the inclusion criteria and were enrolled in this study. Patients with ERMF accounted for 24.3% of the sample (362 patients); a comparable proportion of ERMF patients was maintained between the training set and the test set (24.6% vs 22.7%, p > 0.05). Baseline statistics for both the ERMF and non-ERMF groups are presented in Table 1. Patient characteristics were balanced between the training set (n = 1,193, 80%) and the test set (n = 299, 20%) (Supplementary Table S1).

**TABLE 1:** Demographic and clinical data of the patients.

Feature	ERMF		P
	Non-ERMF (n = 1,130)	ERMF (n = 362)	
Height (cm), Mean ± SD	162.44 ± 5.07	161.81 ± 4.97	0.037
Weight (kg), Mean ± SD	69.51 ± 10.34	69.33 ± 10.08	0.769
BMI (kg/m^2^), Mean ± SD	26.32 ± 3.59	26.44 ± 3.30	0.588
Age (year), Mean ± SD	30.54 ± 3.34	30.63 ± 3.12	0.650
Gestational week (week), M (Q_1_, Q_3_)	39.30 (38.60–40.10)	39.50 (39.10–40.20)	0.001
Intrapartum hemorrhage (mL), M (Q_1_, Q_3_)	200.00 (150.00–400.00)	300.00 (150.00–400.00)	0.004
White blood cell count (10^9^/L), M (Q_1_, Q_3_)	9.30 (8.10–10.50)	9.41 (8.30–11.20)	0.029
Cervical dilatation (cm), M (Q_1_, Q_3_)	2.00 (2.00–3.00)	2.00 (2.00–2.50)	0.775
Neutrophil percentage (%), M (Q_1_, Q_3_)	76.48 (73.90–78.60)	76.62 (74.20–80.57)	0.034
Number of vaginal examinations (n), M (Q_1_, Q_3_)	4.00 (3.00–5.00)	4.00 (3.00–6.00)	0.003
First stage of labor (hour), M (Q_1_, Q_3_)	8.00 (5.87–10.50)	10.00 (8.00–12.96)	<0.001
Second stage of labor (hour), M (Q_1_, Q_3_)	0.33 (0.02–0.75)	0.40 (0.02–0.73)	0.834
Third stage of labor (hour), M (Q_1_, Q_3_)	0.08 (0.07–0.13)	0.08 (0.08–0.12)	0.237
Total duration of labor (hour), M (Q_1_, Q_3_)	8.42 (6.24–11.08)	10.86 (8.45–13.46)	<0.001
Time between rupture of fetal membranes and labor onset (hour), M (Q_1_, Q_3_)	9.11 (5.37–16.35)	10.79 (7.70–16.39)	<0.001
Primiparous woman, n (%)	1,050 (92.92)	352 (97.24)	0.003
Amniotic fluid contamination during membranes rupture, n (%)	64 (5.66)	32 (8.84)	0.032
Amniotic fluid contamination at delivery of the fetus, n (%)	180 (15.93)	72 (19.89)	0.080
Balloons for uterine cervical ripening, n (%)	35 (3.10)	15 (4.14)	0.336
Artificial rupture of membranes, n (%)	668 (59.12)	247 (68.23)	0.002
Gestational diabetes, n (%)	164 (14.51)	46 (12.71)	0.390
Gestational hypertension, n (%)	53 (4.69)	11 (3.04)	0.177
Hypothyroidism in pregnancy, n (%)	126 (11.15)	45 (12.43)	0.506
Giant fetus, n (%)	31 (2.74)	16 (4.42)	0.112
Chorioamnionitis, n (%)	5 (0.44)	8 (2.21)	0.005
Group B *Streptococcus* colonization, n (%)	22 (1.95)	7 (1.93)	0.987
Acute chorioamnionitis, n (%)	5 (0.44)	8 (2.21)	0.005
Hepatitis B in pregnancy, n (%)	7 (0.62)	4 (1.10)	0.557
Immune system diseases, n (%)	24 (2.12)	13 (3.59)	0.118
Preterm premature rupture of membranes, n (%)	285 (25.22)	67 (18.51)	0.009
Pre analgesic prophylactic antimicrobials, n (%)	107 (9.47)	51 (14.09)	0.013
Post analgesic prophylactic antimicrobials, n (%)	210 (18.58)	175 (48.34)	<0.001
Pre analgesic antimicrobials, n (%)	360 (31.86)	115 (31.77)	0.974
Post analgesic antimicrobials, n (%)	338 (29.91)	243 (67.13)	<0.001
Pre analgesic oxytocin, n (%)	864 (76.46)	253 (69.89)	0.012
Post analgesic oxytocin, n (%)	366 (32.39)	165 (45.58)	<0.001
Pre analgesic magnesium sulfate injection, n (%)	52 (4.60)	12 (3.31)	0.293
Post analgesic magnesium sulfate injection, n (%)	31 (2.74)	13 (3.59)	0.407
Pre analgesic diazepam, n (%)	94 (8.32)	33 (9.12)	0.636
Post analgesic diazepam, n (%)	7 (0.62)	2 (0.55)	1.000
Pre analgesic pethidine, n (%)	199 (17.61)	53 (14.64)	0.189
Post analgesic pethidine, n (%)	21 (1.86)	9 (2.49)	0.459
Misoprostol Tablets, n (%)	178 (15.75)	59 (16.30)	0.805
Dinoprostone Suppositories, n (%)	268 (23.72)	101 (27.90)	0.108
Pre analgesic nifedipine, n (%)	83 (7.35)	28 (7.73)	0.806
Post analgesic nifedipine, n (%)	45 (3.98)	12 (3.31)	0.564

(BMI, body mass index; ERMF, epidural-associated maternal fever; M, median; Q1, the first quartile; Q3, the third quartile.).

### 3.2 Feature selection

Table 1 demonstrates that 21 features in the univariate analyses exhibited significant differences between patients with and without ERMF (p < 0.05). Six features with non-zero coefficients were subsequently removed from the LASSO regression. The final fifteen variables incorporated into the ML model were: neutrophil percentage, first stage of labor, amniotic fluid contamination during membrane rupture, artificial rupture of membranes, chorioamnionitis, post analgesic antimicrobials, pre analgesic oxytocin, post analgesic oxytocin, and dinoprostone suppositories (Table 2).

**TABLE 2:** LASSO estimates of selected variables.

Variable	Estimated value
Gestational week	0.111526212
Intrapartum hemorrhage	0.000872844376
Neutrophil percentage	0.104583290
First stage of labor	0.279716503
Primiparous woman	0.0348427924
Amniotic fluid contamination during membranes rupture	0.0480524449
Artificial rupture of membranes	0.0326044562
Chorioamnionitis	0.173171166
Acute chorioamnionitis	0.000000000000000637527975
Pre-analgesic prophylactic antimicrobials	0.00446981574
Post-analgesic prophylactic antimicrobials	0.0715522209
Post-analgesic antimicrobials	0.205517042
Pre-analgesic oxytocin	−0.0575105519
Post-analgesic oxytocin	0.0540198947
Dinoprostone Suppositories	0.0161213136

(LASSO, The least absolute shrinkage and selection operator.).

### 3.3 Model performance

**FIGURE 2:**
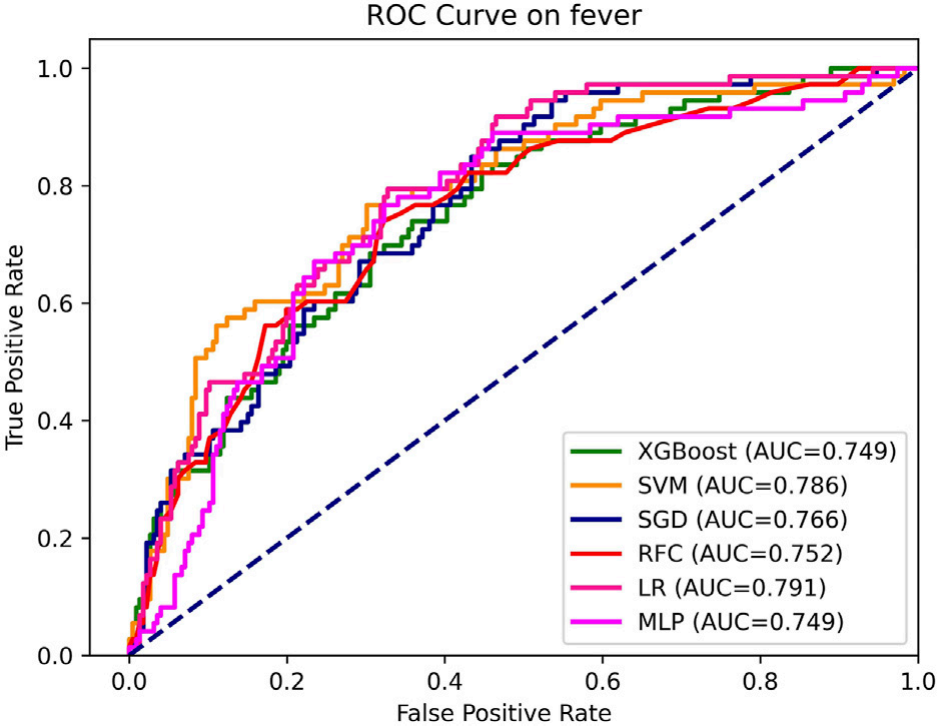
AUC, the area under curve; LR, logistic regression; RFC, random forest classifier; SVM, support vector machine; XGB, extreme gradient boosting; MLP, specifically multi-layer perceptron; SGD, stochastic gradient descent.

The model hyperparameters are presented in Supplementary Table S2. The ROC of each model on the training set is depicted in Figure 2. Table 3 displays the performance metrics for the test set, encompassing AUC, sensitivity, Brier score, calibrated slope, and calibrated intercept.

**TABLE 3:** Discrimination and calibration of each machine learning algorithms on the testing set.

Model	AUC	Accuracy	Precision	Sensitivity	F1 score	Intercept	Slope	Brier
LR	0.791	0.70	0.44	0.79	0.57	−0.062	0.715	0.193
SVM	0.786	0.79	0.57	0.59	0.58	0.085	0.428	0.155
RFC	0.752	0.69	0.43	0.74	0.54	0.012	0.787	0.156
XGBoost	0.749	0.69	0.41	0.68	0.52	0.109	0.537	0.176
SGD	0.766	0.74	0.47	0.63	0.54	0.120	0.867	0.184
MLP	0.749	0.71	0.44	0.70	0.54	0.231	0.306	0.184

(AUC, area under curve of receiver operating characteristic; LR, logistic regression; RFC, random forest classifier; SVM, support vector machine; XGB, extreme gradient boosting; MLP, specifically multi-layer perceptron; SGD, stochastic gradient descent).

As depicted in Table 3 and Figure 2, the discriminative performance was observed in LR (AUC, 0.791), SVM (AUC, 0.786), SGD (AUC, 0.766), RFC (AUC, 0.752), XGB (AUC, 0.749), and MLP (AUC, 0.749) on the testing set. DeLong’s test revealed a statistically significant difference in AUC between the LR model and the SGD model, (P < 0.05). No statistically significant differences were observed among the remaining models (Supplementary Table S3).

**FIGURE 3:**
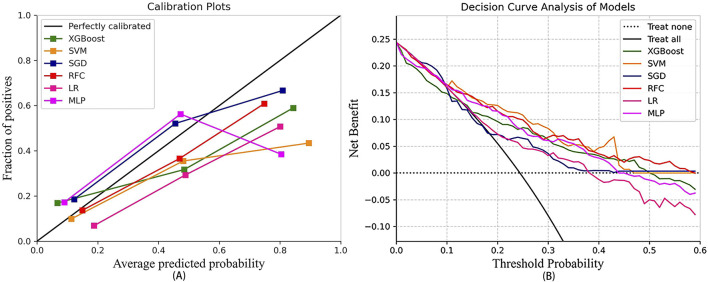
The calibration curve of the machine learning models **(A)** and decision curve analysis of the machine learning models **(B)**. LR, logistic regression; RFC, random forest classifier; SVM, support vector machine; XGB, extreme gradient boosting; MLP, specifically multi-layer perceptron; SGD, stochastic gradient descent.

In the test set, Brier scores ranged from 0.155 to 0.193, calibrated slopes ranged from 0.306 to 0.867, and calibrated intercepts ranged from −0.062 to 0.109 (Figure 3A; Table 3). Decision curve analyses demonstrated that the SVM and RFC models exhibited a higher net benefit and a default strategy of treating all patients or no patients compared to other ML models (Figure 3B).

Considering all factors, the LR model’s AUC was statistically different from that of the RFC, and the LR also demonstrated superior calibration compared to the SVM (Brier score, 0.193; calibration slope, 0.715; calibration intercept, −0.062). Consequently, the LR model was selected as the predictive model.

### 3.4 Development a nomogram for the prediction of ERMF with the LR model

**FIGURE 4:**
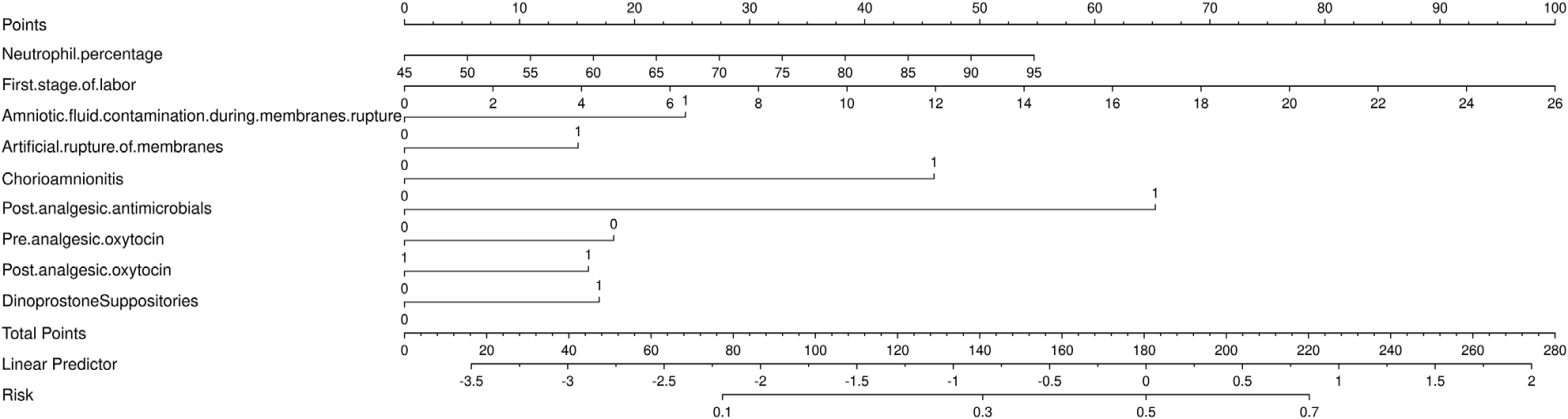
Nomogram for the prediction of epidural-associated maternal fever after Patient-controlled epidural analgesia.

To predict ERMF, we constructed an LR-based nomogram. The nomogram identifies eight key predictors for ERMF prediction: neutrophil percentage, first stage of labor, amniotic fluid contamination during membrane rupture, artificial rupture of membranes, chorioamnionitis, post-analgesic antimicrobials, pre-analgesic oxytocin, post-analgesic oxytocin, and dinoprostone suppositories (refer to Figure 4).

## 4 Discussion

The etiology of maternal fever induced by epidural labor analgesia remains uncertain, and the application of specific preventive or therapeutic measures for epidural-associated maternal fever presents challenges. Several studies have investigated factors influencing the incidence of ERMF to varying degrees, with the aim of identifying effective preventive strategies (Ren et al., 2021; Wang et al., 2023; Yin and Hu, 2022). Machine learning has seen extensive application in obstetrics and gynecology in recent years. However, its utilization in the context of epidural labor analgesia and its association with ERMF remains unexplored.

In this retrospective study, we compared the performance of traditional binomial logistic regression with machine learning algorithmic prediction models for forecasting ERMF in pregnant women who underwent PCEA, utilizing real-world research data. The results indicated that the LR model achieved the highest AUC among all tested models. Additionally, the application of a nomogram to depict pertinent factors influencing ERMF, including neutrophil percentage, first stage of labor, Amniotic fluid contamination during membranes rupture, artificial rupture of membranes, chorioamnionitis, post-analgesic antimicrobials, pre analgesic oxytocin, post analgesic oxytocin, and dinoprostone suppositories, underscores the importance of further analyzing these results. Furthermore, the AUC across all examined models ranged from 0.749 to 0.791, indicating that several models in this study exhibit strong generalization capabilities within this cohort. In this study, we found no evidence to support that machine learning-based ERMF prediction models have better performance than logistic regression-based models.

The LR model exhibited superior sensitivity but lower accuracy compared to the machine learning model, achieving 44% precision. The LR model sacrificed some accuracy to achieve improved AUC and sensitivity. After careful consideration, we opted for the predictive model based on LR analysis for three key reasons. ERMF has a prevalence of approximately 20% (Fusi et al., 1989), and the dataset suffers from severe imbalance. It is important to note that a predictive model can achieve high accuracy [(TP + TN)/(TP + TN + FP + FN)] if it simply predicts that all patients do not have ERMF. Hence, when comparing different models, accuracy is not the primary concern. Second, for ERMF patients, sensitivity holds greater importance than accuracy. The development of ERMF in a woman can lead to severe adverse pregnancy outcomes. Third, screening these variables is more straightforward when visualized in a nomogram. All variables are interpretable and quantifiable, eliminating the “black box” nature of machine learning. This outcome is unsurprising, as previous evidence has demonstrated that machine learning does not outperform logistic regression in clinical prediction models (Christodoulou et al., 2019).

Our study revealed an elevated neutrophils percentage and an extended first stage of labor, both of which are associated with ERMF, which is consistent with previous studies (Zhao et al., 2022; Curtin et al., 2015). This may be due to the fact that extended labor may deplete physical resources, potentially compromising immunity and elevating the risk of infection (Goetzl et al., 2001). Also, previous studies have confirmed a higher risk of fever in women with placental neutrophilic inflammation (Sultan and Segal, 2020). Furthermore, instances of epidural fever in women opting for labor analgesia are often linked to chorioamnionitis and placental inflammation (Sharma, 2000). Caution is warranted when interpreting the relationship between ERMF and elevated neutrophil percentages in this study since these neutrophil percentages represent the most recent tests conducted before PCEA. Previous study have demonstrated a lack of significant correlation between the development of puerperal fever and the levels of inflammatory factors during pregnancy (Arce et al., 2019), which contradicts the findings of the present study. Consequently, further studies are required to investigate the relationships among maternal fever, maternal inflammation levels, and potential sources of inflammation. It is yet to be determined whether fever during labor is linked to prenatal inflammation levels or if it is triggered by events during labor that amplify the maternal inflammatory response. Additionally, our study identified amniotic fluid contamination during membrane rupture, artificial membrane rupture, and post-analgesic antimicrobials as independent risk factors for ERMF, consistent with previous research on risk factors for intrapartum fever (Jiang et al., 2021; Maayan-Metzger et al., 2006; Kim et al., 2021). Interestingly, the use of oxytocin before analgesia acted as a protective factor against ERMF, while its use after the initiation of analgesia emerged as a risk factor. Previous studies have indeed linked oxytocin use to an elevated risk of ERMF (Chang et al., 2023). However, given the scarcity of research regarding the timing of oxytocin use, further investigation is needed to fully understand its impact on ERMF.

To the best of our knowledge, this study is the first to use a machine model for predicting ERMF and to contrast it with a traditional regression model using extensive data on pregnancy history, clinical assessments, and pregnancy biochemical variables. However, there are certain limitations. Firstly, it was a retrospective study with inherent shortcomings, including inadequate documentation of maternal and fetal information by healthcare providers, resulting in incomplete data collection. The study’s sample size is small, and it is a single-center study. To enhance the study’s robustness, a larger, multi-center study should be considered in the future. Despite ERMF having an incidence of approximately 20%, maternal fever has significant adverse implications for maternal safety and neonatal wellbeing. Women with maternal fever are at an increased likelihood of receiving antibiotics, undergoing cesarean sections, and experiencing low Apgar scores, respiratory distress, hypotonia, neonatal convulsions, and neonatal encephalopathy, all of which have grave consequences (Dior et al., 2016; Towers et al., 2017). Hence, there is merit in developing predictive models for pregnant women with ERMF. Prospective studies could offer additional variables for prediction and enable a more precise assessment of ERMF. Subsequent prospective studies are necessary. Secondly, this study lacked external validation of the model, which may limit the broad application of the results. Therefore, external validation will be carried out in a multicenter prospective study. Lastly, the performance of several models in our study was only moderate, with an AUC of <80% for all models. Consequently, the validity of using these predictive models remains a subject of debate.

## 5 Conclusion

In summary, we developed six prediction models for ERMF using LR, RF, SVM, XGBoost, GBM, and MLP. Although the studied machine learning models can predict ERMF, they do not surpass the current gold standard logistic regression, which exhibits superior AUC, sensitivity, and interpretability. Additionally, while predictive models enable preoperative risk assessment and treatment decisions, they still necessitate clinical expertise. Furthermore, identifying predictors of specific factors, such as medication considerations and comorbid conditions, in predictive models could enhance perioperative care. This, in turn, enables timely interventions by physicians and nurses, ultimately leading to improved surgical outcomes.

## Data Availability

The raw data supporting the conclusions of this article will be made available by the authors, without undue reservation.
