# Supplementary material for: Machine learning algorithms to predict epidural-related maternal fever: a retrospective study
Source: Front Pharmacol. 2025 Jun 11;16:1614770. doi: 10.3389/fphar.2025.1614770 (PMC12187650; doi:10.3389/fphar.2025.1614770)
Supplement: Supplementary file 1 [file Supplementaryfile1.docx]

**Supplemental Tables**

**Table S1** Demographics and clinical characteristics of the training and testing sets

| **Variable** | **Total (n = 1492)** | **train_set (n = 1193)** | **test_set (n = 299)** | **P** |
| --- | --- | --- | --- | --- |
| Height (cm), Mean ± SD | 162.29 ± 5.06 | 162.19 ± 5.03 | 162.67 ± 5.14 | 0.141 |
| Weight(kg), Mean ± SD | 69.47 ± 10.28 | 69.61 ± 10.46 | 68.92 ± 9.50 | 0.302 |
| BMI (kg/m²), Mean ± SD | 26.35 ± 3.52 | 26.43 ± 3.60 | 26.02 ± 3.16 | 0.069 |
| Age (year), Mean ± SD | 30.56 ± 3.29 | 30.58 ± 3.32 | 30.49 ± 3.17 | 0.655 |
| Gestational week (week), M (Q₁, Q₃) | 39.30 (39.00 - 40.10) | 39.30 (39.00 - 40.10) | 39.30 (38.80 - 40.10) | 0.940 |
| Intrapartum hemorrhage (ml), M (Q₁, Q₃) | 200.00 (150.00 - 400.00) | 200.00 (150.00 - 400.00) | 250.00 (150.00 - 415.00) | 0.227 |
| White blood cell count (10^9/L  ), M (Q₁, Q₃) | 9.32 (8.10 - 10.60) | 9.38 (8.10 - 10.70) | 9.16 (8.10 - 10.25) | 0.081 |
| Cervical dilatation(cm), M (Q₁, Q₃) | 2.00 (2.00 - 3.00) | 2.00 (2.00 - 3.00) | 2.00 (2.00 - 2.75) | 0.297 |
| Neutrophil percentage(%), M (Q₁, Q₃) | 76.50 (74.00 - 79.17) | 76.51 (74.00 - 79.00) | 76.47 (74.00 - 79.50) | 0.822 |
| Number of vaginal examinations (n), M (Q₁, Q₃) | 4.00 (3.00 - 6.00) | 4.00 (3.00 - 5.00) | 4.00 (3.00 - 6.00) | 0.499 |
| First stage of labor (hour), M (Q₁, Q₃) | 8.50 (6.00 - 11.00) | 8.50 (6.25 - 11.00) | 9.00 (6.00 - 11.50) | 0.478 |
| Second stage of labor (hour), M (Q₁, Q₃) | 0.35 (0.02 - 0.73) | 0.33 (0.02 - 0.72) | 0.42 (0.02 - 0.79) | 0.141 |
| Third stage of labor (hour), M (Q₁, Q₃) | 0.08 (0.07 - 0.13) | 0.08 (0.07 - 0.13) | 0.08 (0.07 - 0.13) | 0.207 |
| Total duration of labor (hour), M (Q₁, Q₃) | 9.08 (6.73 - 11.63) | 8.92 (6.73 - 11.58) | 9.45 (6.77 - 12.09) | 0.325 |
| Time between rupture of fetal membranes and laboronset (hour), M (Q₁, Q₃) | 9.69 (5.88 - 16.35) | 9.78 (5.98 - 16.40) | 9.18 (5.50 - 15.80) | 0.234 |
| Fever, n (%) | 362 (24.26) | 294 (24.64) | 68 (22.74) | 0.493 |
| Primiparous woman, n (%) | 1402 (93.97) | 1119 (93.80) | 283 (94.65) | 0.580 |
| Amniotic fluid contamination during membranes rupture, n (%) | 96 (6.43) | 81 (6.79) | 15 (5.02) | 0.264 |
| Amniotic fluid contamination at delivery of the fetus, n (%) | 252 (16.89) | 202 (16.93) | 50 (16.72) | 0.931 |
| Balloons for uterine cervical ripening, n (%) | 50 (3.35) | 36 (3.02) | 14 (4.68) | 0.153 |
| Artificial rupture of membranes, n (%) | 915 (61.33) | 720 (60.35) | 195 (65.22) | 0.122 |
| Gestational diabetes, n (%) | 210 (14.08) | 167 (14.00) | 43 (14.38) | 0.865 |
| Gestational hypertension, n (%) | 64 (4.29) | 51 (4.27) | 13 (4.35) | 0.956 |
| Hypothyroidism in pregnancy, n (%) | 171 (11.46) | 139 (11.65) | 32 (10.70) | 0.645 |
| Giant fetus, n (%) | 47 (3.15) | 33 (2.77) | 14 (4.68) | 0.090 |
| Chorioamnionitis, n (%) | 13 (0.87) | 11 (0.92) | 2 (0.67) | 0.942 |
| Group B Streptococcus colonization, n (%) | 29 (1.94) | 25 (2.10) | 4 (1.34) | 0.396 |
| Acute chorioamnionitis, n (%) | 13 (0.87) | 11 (0.92) | 2 (0.67) | 0.942 |
| Hepatitis B in pregnancy, n (%) | 11 (0.74) | 11 (0.92) | 0 (0.00) | 0.198 |
| Immune system diseases, n (%) | 37 (2.48) | 30 (2.51) | 7 (2.34) | 0.863 |
| Preterm premature rupture of membranes, n (%) | 352 (23.59) | 289 (24.22) | 63 (21.07) | 0.251 |
| Pre analgesic prophylactic antimicrobials, n (%) | 158 (10.59) | 127 (10.65) | 31 (10.37) | 0.889 |
| Post analgesic prophylactic antimicrobials, n (%) | 385 (25.8) | 297 (24.90) | 88 (29.43) | 0.109 |
| Pre analgesic antimicrobials, n (%) | 475 (31.84) | 383 (32.10) | 92 (30.77) | 0.658 |
| Post analgesic antimicrobials, n (%) | 581 (38.94) | 455 (38.14) | 126 (42.14) | 0.205 |
| Pre analgesic oxytocin, n (%) | 1117 (74.87) | 894 (74.94) | 223 (74.58) | 0.899 |
| Post analgesic oxytocin, n (%) | 531 (35.59) | 416 (34.87) | 115 (38.46) | 0.246 |
| Pre analgesic magnesium sulfate injection, n (%) | 64 (4.29) | 51 (4.27) | 13 (4.35) | 0.956 |
| Post analgesic magnesium sulfate injection, n (%) | 44 (2.95) | 37 (3.10) | 7 (2.34) | 0.487 |
| Pre analgesic diazepam, n (%) | 127 (8.51) | 101 (8.47) | 26 (8.70) | 0.899 |
| Post analgesic diazepam, n (%) | 9 (0.6) | 4 (0.34) | 5 (1.67) | 0.024 |
| Pre analgesic pethidine, n (%) | 252 (16.89) | 196 (16.43) | 56 (18.73) | 0.343 |
| Post analgesic pethidine, n (%) | 30 (2.01) | 23 (1.93) | 7 (2.34) | 0.649 |
| Misoprostol Tablets, n (%) | 237 (15.88) | 180 (15.09) | 57 (19.06) | 0.093 |
| Dinoprostone Suppositories, n (%) | 369 (24.73) | 297 (24.90) | 72 (24.08) | 0.770 |
| Pre analgesic nifedipine, n (%) | 111 (7.44) | 91 (7.63) | 20 (6.69) | 0.580 |
| Post analgesic nifedipine, n (%) | 57 (3.82) | 52 (4.36) | 5 (1.67) | 0.030 |

BMI, Body mass index; ERMF, epidural-associated maternal fever; M, median; Q1, the first quartile; Q3, the third quartile.

**Table S2** Hyper-parameters of each model

| Model | Hyper-parameters | parameter values |
| --- | --- | --- |
| LR | C | 1.0 |
|  | class_weight | balanced |
| RFC | n_estimators | 100 |
|  | min_samples_split | 2 |
|  | min_samples_leaf | 1 |
|  | class_weight | balanced |
| SVM | C | 1.0 |
|  | gamma | 0.01 |
|  | class_weight | balanced |
| XGB | n_estimators | 100 |
|  | gamma | 0 |
|  | max_depth | 6 |
| SGD | class_weight | balanced |
|  | penalty | 12 |
|  | alpha | 0.0001 |
|  | l1_ratio | 0.15 |
| MLP | Hidden layers | 100-100-100 |
|  | class_weight | balanced |
|  | learning rate | 0.001 |
|  | beta_1 | 0.9 |
|  | beta_2 | 0.999 |
|  | alpha | 0.0001 |

LR, logistic regression; RFC, random forest classifier; SVM, support vector machine; XGB, extreme gradient boosting; MLP, specifically multi-layer perceptron; SGD, stochastic gradient descent.

**Table S3** The *p*-values of pairwise comparisons of AUCs on testing set for different models with Delong test

| Model | RFC | SVM | XGB | SGD | MLP |
| --- | --- | --- | --- | --- | --- |
| LR | 0.0708 | 0.7682 | 0.1089 | 0.00133 | 0.1177 |
| RFC |  | 0.1185 | 0.8891 | 0.5446 | 0.8973 |
| SVM |  |  | 0.1442 | 0.3669 | 0.1009 |
| XGB |  |  |  | 0.5504 | 0.9854 |
| SGD |  |  |  |  | 0.5454 |

The significant difference between AUCs is defined as p-value < 0.05. Abbreviations: AUC, the area under receiver operating characteristic curve; LR, logistic regression; RFC, random forest classifier; SVM, support vector machine; XGB, extreme gradient boosting; MLP, specifically multi-layer perceptron; SGD, stochastic gradient descent.
